# Postpartum infective endocarditis with *Enterococcus faecalis* in Japan: a case report

**DOI:** 10.1186/s13256-017-1494-x

**Published:** 2017-11-17

**Authors:** Miku Tamura, Mitsutaka Shoji, Ken Fujita, Shohei Nakamura, Yurika Takahashi, Yurika Suzuki, Mika Asakura, Shun Kimizuka, Makiko Sasaki, Katsuya Sugawara

**Affiliations:** Department of Pharmacy, Funabashi Municipal Medical Center, 1-21-1 Kanasugi, Funabashi city, Chiba prefecture Japan

**Keywords:** *Enterococcus faecalis*, Infective endocarditis, Postpartum period

## Abstract

**Background:**

The clinical characteristics of infective endocarditis include the presence of predisposing cardiac disease, a history of illegal drug use, and high morbidity in the elderly. Only a few cases of the disease after delivery have been reported in the literature. We describe here a first case of enterococcal postpartum infective endocarditis without underlying disease in Japan.

**Case presentation:**

We report the case of a 31-year-old Japanese woman with postpartum infective endocarditis by *Enterococcus faecalis*. She had no significant medical history or any unusual social history. After emergency surgery for severe mitral regurgitation and antimicrobial treatment for 6 weeks, she was discharged from our hospital and is now being monitored at an out-patient clinic.

**Conclusions:**

We encountered a case of *Enterococcus faecalis* infective endocarditis that occurred in the native valve of a postpartum healthy woman. Although the pathogenesis of this case remains unclear, it could be due to bacteremia arising from the administration of prophylactic broad-spectrum antibiotics used for cesarean section. Previous use of cefotiam and urinary catheter insertion may be risk factors for nosocomial enterococcal bacteremia in this case.

## Background

Infective endocarditis (IE) is uncommon but causes high mortality. Differences in the causative microorganism of IE depend on backgrounds across different age groups (that is, pre-existing valvular heart disease) and countries [1–3]. *Streptococcus* species (40%) and *Staphylococcus* species (40%) account for 80% of the cases of IE, and *Enterococcus* species are responsible for up to only 10% [1, 4–6]. Identified risk factors of IE are intravenous drug use, congenital heart disease, and chronic rheumatic heart disease [1–3].

Enterococci are usually regarded as indigenous flora of the intestinal tract, oral cavity, and the genitourinary tract of humans. Among the elderly who have immune system compromise, *Enterococcus* species cause approximately 20% of nosocomial IE [2]. Our understanding of IE in pregnancy and the postpartum period is limited by extremely low numbers of reported incidents [7], especially regarding incidents of IE in the postpartum period due to *Enterococcus* species.

We report a case of IE caused by *Enterococcus faecalis* in a 31-year-old woman after cesarean section. To the best of our knowledge, this is the first reported case of postpartum *Enterococcus faecalis* IE described in the literature.

## Case presentation

A 31-year-old Japanese woman presented to our hospital with approximately a 2-month history of intermittent fever and night sweats. She had delivered a baby by cesarean section in a local hospital 3 months prior to admission. These symptoms appeared within a few weeks of her labor. A month prior to admission, she reported edema of her lower limbs and dizziness. She was transferred to our hospital for general fatigue, chest pain, and dyspnea. She had no significant medical history and no relevant history of dental procedures, exposure to animals, travel, or drug abuse. She had no congenital heart disease; this was checked by echocardiography in former hospital for cesarean section.

Her body temperature was 37.8 °C, respiratory rate was 24 breaths/minute, systolic blood pressure was 70 mmHg, and she had pale conjunctivae without petechiae and no signs of Osler nodes or Janeway lesions. A laboratory workup revealed a white blood cell (WBC) count of 8.5 × 10^9^/L, hemoglobin 9.9 g/dL, and serum creatinine (Scr) 0.83 mg/dL; her C-reactive protein (CRP) was elevated at 20.5 mg/dL.

Transthoracic echocardiography revealed a mobile, 20 × 15 mm vegetation attached to the anterior mitral valve; mitral valve replacement with a prosthetic valve was urgently performed because of the severe mitral regurgitation and worsening symptoms of heart failure.

**Fig. 1:**
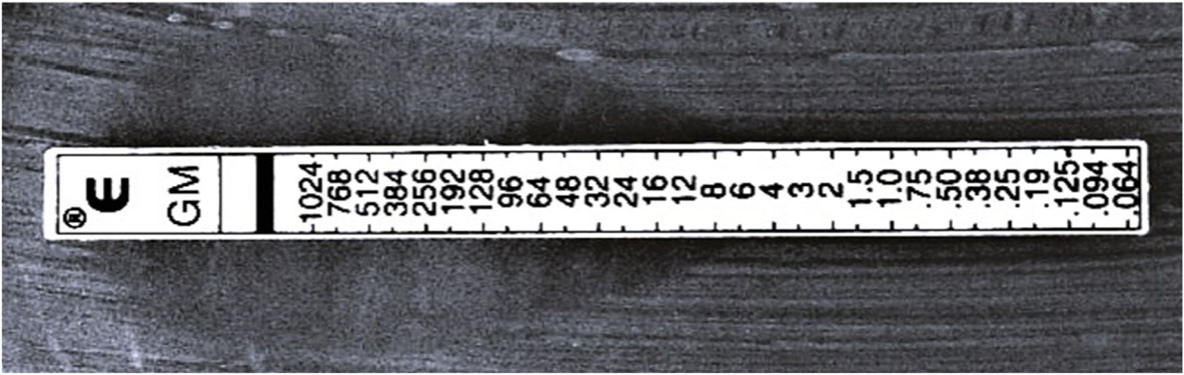
The susceptibility of *Enterococcus faecalis* by E-test. *Enterococcus faecalis* in this case did not show high-level gentamicin resistance

**Fig. 2:**
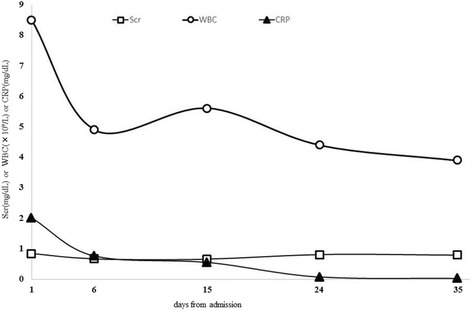
Clinical course of the patient. Transitive graph showing the clinical course of the patient from day 1 (admission day and start of treatment) to day 35 (1 week before antibiotic treatment ended). Abbreviations: *CRP* C-reactive protein, *Scr* serum creatinine, *WBC* white blood cell

Two sets of blood cultures drawn on admission and valve tissue culture yielded *Enterococcus faecalis*. The *Enterococcus faecalis* in this case was susceptible to benzylpenicillin G, amino-benzyl penicillin (ampicillin), and vancomycin (Table 1). The gentamicin minimum inhibitory concentration (MIC) was 8 mg/L, which was not high-level resistance as detected by E-test® strips (AB Biodisk, Sweden; Fig. 1). The diagnosis of IE was confirmed with two of the major items of the modified Duke criteria: positive blood culture and echocardiogram with an oscillating intracardiac mass on the valve. She was placed on antimicrobial therapy with intravenously administered ampicillin (2 g every 4 hours) and gentamicin (1 mg/kg every 8 hours) for 6 weeks. During the 6-week treatment period, we monitored WBC, Scr, and CRP (Fig. 2). We also checked the serum concentration of gentamicin in pre-dosing 0.4 mg/L on the fifth day after initiation of gentamicin therapy. Her renal function, with a creatinine clearance of nearly 100 mL/minute at the end of the treatment period, did not deteriorate.

**Table 1:** The susceptibility of *Enterococcus faecalis* (microdilution method)

	MIC	susceptibility
PCG	2	S
ABPC	2	S
VCM	2	S
RFP	>2	R

Abbreviations: *ABPC* amino-benzyl penicillin (ampicillin), *MIC* minimum inhibitory concentration, *PCG* benzylpenicillin G, *R* resistant, *RFP* rifampicin, *S* susceptible, *VCM* vancomycin

Vegetation was not detected by transthoracic echocardiography after the mitral valve replacement. She was discharged from our hospital without any complications on the 42nd day of hospitalization. She did not have any sequelae related to the disease 19 months after discharge.

## Discussion

Enterococcal endocarditis is estimated to represent only 10% of all endocarditis cases. Enterococcal endocarditis has been linked to genitourinary instrumentation and biliary portals, and unlike *Staphylococcus* endocarditis, valvular heart disease is not always a prerequisite.

Enterococci, including *Enterococcus faecalis*, are one of the normal bacterial flora of the intestine, the female genital tract, and the dental cavity [8]. Our patient was a housewife and did not receive periodic medical (including dental) treatment. This suggests that the intestinal flora, vaginal flora, and periodontal pockets might have served as potential reservoirs for the post-delivery endocarditis in this patient.

Prophylactic antimicrobials administration in cesarean sections can reduce the incidence of post-delivery infection, including fever, wound infection, and pelvic abscess [9]. The guidelines of the American College of Obstetricians and Gynecologists, Infectious Disease Society of America, and the Japanese guidelines recommend the use of narrow-spectrum antimicrobials for prophylaxis, avoiding broad-spectrum antimicrobials [10, 11]. The Cochrane review showed that ampicillin and first-generation cephalosporins have similar efficacies for postoperative infection after cesarean sections [9], and broad-spectrum antimicrobials did not show any benefit. In this case, at the former hospital, despite the absence of a wound infection, the prophylactic antibiotic cefotiam, a second-generation cephalosporin, used against most common Gram-positive and Gram-negative pathogens, was administered 1 g every 12 hours for 3 days after cesarean section. Previous reports have implicated broad-spectrum antibiotics such as moxalactam in the development of enterococcal superinfection [12, 13]. Siesing *et al*. [14] reported a correlation between cefuroxime, a second-generation cephalosporin, consumption, and the rising incidence of enterococci in bone and soft tissue biopsies. The most frequent site of superinfection was the urinary tract of which the incidence exceeded 5% [12]. A similar observational study has suggested that the use of second-generation and third-generation cephalosporins was a risk factor for nosocomial enterococcal bacteremia (NEB) [15]. The mechanisms of enterococcal superinfections have not been elucidated in detail. *Enterococcus* colonization and overgrowth appears to be linked to a rise of superinfection. The overgrowth of enterococci in the cervicovaginal microflora after three 2 g cefoxitin [16], a second-generation cephalosporin, and one 1 g ceftriaxone [17], a third-generation cephalosporin, have occurred. NEB have well-known risk factors including indwelling urinary catheter beyond 24 hours and surgery. Our patient did not have a history of recurrent urinary tract infection (UTI) and urine cultures were negative on the admission day with IE. Urinary catheter insertion was performed for 2 days during the hospitalization for childbirth. Previous use of cefotiam and urinary catheter insertion may be risk factors for NEB in this case [18].

Enterococcal endocarditis results in considerable mortality and cure rates of 72 to 79% are reported using standard therapy. Ampicillin monotherapy often results in clinical failures; therefore, aminoglycosides are added, because this combination enhances the bactericidal activity *in vitro*. Naturally, all enterococci display low aminoglycoside resistance; however, synergy is maintained with cell wall-active agents. High-level resistance to aminoglycosides abolishes the synergistic bactericidal activity of aminoglycosides in combination with cell-wall-active agents such as ampicillin, which are important in the treatment of severe enterococcal infections such as endocarditis. Enterococcal strains resistant to high levels of gentamicin (MIC > 512 mg/L) have been increasing among *Enterococcus faecalis* and *Enterococcus faecium*. In a study by Fernandez-Hidalgo *et al*., 26% of 272 patients were infected by high-level aminoglycoside-resistant strains [19]. Therefore, a gentamicin-containing regimen would not be effective for these patients. Gentamicin-associated nephrotoxicity may complicate [20] a standard 4-week to 6-week course regimen and could result in serious, life-threating complications, such as renal failure, requiring hemodialysis. To prevent renal failure, pre-dose gentamicin levels must be monitored. The American Heart Association guidelines recommend a pre-dose gentamicin concentration of less than 1 mg/L [21]. In this case, before applying a gentamicin-containing regimen, we confirmed that the causative *Enterococcus faecalis* did not display high-level gentamicin resistance. According to the results, we adjusted the optimal dosing to a pre-dose level of less than 1 mg/L, offering the highest efficacy without causing renal dysfunction.

In this case, 4 months’ delay to diagnosis of IE let the mitral vegetation develop larger before the urgent surgery. Before admission, two primary care physicians had seen the patient and considered that she had a cold. Since no abnormal laboratory data were obtained from the patient, they prescribed antipyretic routinely without recognizing the fever to be of unknown origin, including the possibility of IE. It is hard to suspect because she had no medical history of conventional risk factors of IE.

In our clinical setting, we examined the high-level gentamicin resistance to ensure effectiveness of the ongoing antimicrobial treatment, and did not pay enough attention to check abdominal computed tomography (CT), colon fiber examination, and serum antistreptolysin O titers. A dental examination revealed no abnormalities. Our investigation was insufficient to evaluate multiple factors of the entry site due to her bacteremia.

In considering her clinical history, the nonspecific symptoms of IE started after cesarean section. The origin of the bacteremia was naturally elicited during the perioperative processes. Our report suggested the indwelling urinary catheter and the prophylactic inappropriate antibiotics can be risk factors of IE in an ostensibly healthy postpartum woman.

## Conclusions

We report here a rare case of postpartum IE caused by *Enterococcus faecalis* in a healthy woman. The pathogen is usually virulent to the elderly with immune deficiencies, and it often causes nosocomial infections. In this case, prophylactic cefotiam for the cesarean section might have selected the pathogen, leading to bacteremia and IE.
